# The Impact of Sleep and Mental Health on Working Memory and Academic Performance: A Longitudinal Study

**DOI:** 10.3390/brainsci12111525

**Published:** 2022-11-10

**Authors:** Abeer F. Almarzouki, Rahaf L. Mandili, Joud Salloom, Lujain K. Kamal, Omimah Alharthi, Samah Alharthi, Nusaiba Khayyat, Alaa M. Baglagel

**Affiliations:** 1 Department of Physiology, Faculty of Medicine, King Abdulaziz University, Jeddah 21589, Saudi Arabia; 2 Faculty of Medicine, King Abdulaziz University, Jeddah 21589, Saudi Arabia

**Keywords:** sleep, working memory, academic performance, mental health

## Abstract

Sleep and mental health can affect cognition and academic performance. The present study aimed to investigate the relationships between sleep, mental health, working memory, and academic performance. We collected demographic data from university students during the non-academic summer period and the academic term. We also measured academic performance (GPA), sleep (PSQI), depression (PHQ-9), anxiety (GAD-7), and disordered social media use (SMDS). Working memory was assessed by the Cambridge Neuropsychological Test Automated Battery (CANTAB). We assessed 83 students (42.2% male) with a mean age of 21 years. Compared to the non-academic summer period, students had significantly worse sleep and distress scores in the academic term. Anxiety, depression, and distress scores were significantly correlated with worse sleep quality. Despite worse mental health and sleep in the academic term, working memory improved compared to the non-academic summer period and was also correlated with a higher GPA. However, a higher GPA was significantly associated with longer sleep latency, increased sleep disturbances, and increased use of sleep medication. Students experiencing poor sleep suffered from poor mental health, although they maintained high GPA and working memory scores. Cognitive resilience, including higher working memory, may mask poor sleep quality and mental health among university students.

## 1. Introduction

The university years can be a challenging life stage for students [1]. Many students struggle to juggle academic demands and study–life balance [2], in addition to worries about securing a job in a competitive market [3]. Such difficulties are particularly true in challenging specialties such as medical or health-based fields [4]. Hence, this population of young people is at a risk of developing mental challenges during their university years [5]. These concerns have driven a global interest among health, research, and academic professionals in the investigation of such challenges in the hope of minimizing the impact of these stressors on student well-being [6,7].

Sleep is one factor that has deteriorated in recent years, especially in this age group and largely due to changes in behavioral and social factors [8]. University students typically shift to irregular sleep patterns as a result of academic schedules and deadlines [9]. Adequate sleep is a vital component of human beings’ survival and plays a key role in an individual’s physical and mental health [10]. In addition, sleep has a crucial effect on optimal cognitive functioning, including attention, working memory and perception [11]. Working memory is a type of memory that holds task-relevant information while the brain performs other mental tasks [12]. We focused specifically on working memory because it is a vital cognitive function in the process of learning [13]. Working memory is a strong predictor of academic performance, stronger than IQ level [14]. Students with working memory defects are more prone to academic failure [15]. Furthermore, working memory is vulnerable to sleep disruption [16].

Furthermore, sleep has an essential role in emotional regulation and mental health [17]. Previous research has demonstrated that sleep disturbances may lead to psychiatric disorders such as depression, anxiety, and psychological distress even among previously healthy individuals [18].

Numerous studies worldwide have explored the relationship between academic performance and sleep. Findings from these studies generally support a positive association between better sleep quality and higher academic performance [19,20,21], although some studies suggest that this association could be negative [22,23,24] or does not exist [25]. These discrepancies could be explained by incomplete investigation of the relevant factors which potentially influence the relationship between sleep and academic performance, such as mental health [26]. Furthermore, many of these studies relied solely on grade point average (GPA) or exam scores, reported by the students, as a measure of academic ability. Finally, most of these studies had a single cross-sectional design that assessed only a single record of participant behaviors, primarily during the academic term, which limits our understanding of how participant performance and behavior changes in response to attending university.

In the current study, we aimed to account for affective factors, namely depression, anxiety and stress that may impact academic performance. In addition, we provided an objective measure of cognitive ability, not just a behavioral academic outcome (GPA). Investigating these two measures, mental wellbeing and an objective measure of cognitive ability, will provide a better perspective on the relationship between sleep and academic performance, specifically in a longitudinal study design.

## 2. Methodology

### 2.1. Ethical Statement

The institutional review board at King Abdulaziz University Hospital (KAUH) provided ethical approval after reviewing the proposal for this study. All participants agreed to be a part of the study after reading the information sheet and signing the consent form. The right of withdrawal at any point in the study was clearly explained to each participant, and all participant responses were anonymized.

### 2.2. Participants

We recruited the study participants by posting messages on social media (i.e., WhatsApp) that briefly explained the study. Participants were screened according to the study inclusion and exclusion criteria. We included male and female university students aged 18 years and older but not those who were in their final year of study. We excluded participants who had previously been diagnosed with chronic medical or physiological conditions. No compensation of any kind was given to the participants. After applying the inclusion and exclusion criteria, the total number of participants enrolled was 83. All participants signed electronic consent forms and were informed of the right to withdraw at any time during the study period. Prior to the start of the study, the researchers explained to the participants the study procedure (see image below), the purpose of the assessment tools used, and how they would be utilized. The researchers received confirmation from the participants that they understood what had been explained to them before filling out the questionnaire and participating in the study procedures and before taking the Cambridge Neuropsychological Test Automated Battery (CANTAB).

### 2.3. Study Design

**Figure 1:**
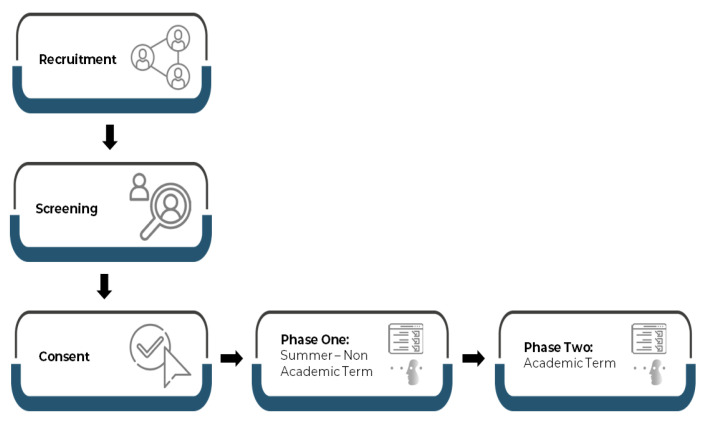
Study Design.

The present study was a longitudinal study designed to obtain and analyze data from university students in the non-academic summer period and during the academic term. All participants were enrolled at King Abdulaziz University (KAU), Jeddah, Saudi Arabia. The first data collection phase took place in the middle of the summer vacation (June 2021), and the second phase was in the middle of the first academic semester (November 2021). Working memory was assessed by CANTAB. Sleep, anxiety, depression, distress scores, social media use were measured by validated scales via digital questionnaires (Figure 1). All students were English-speaking and studying an English-based curriculum, and all questionnaires were given in English. Participants met with researchers in a quiet environment on the KAU campus. All participants received the same instructions and were trained on the device and the use of CANTAB. All participants subsequently took part in a practice CANTAB trial before the actual CANTAB trials began.

### 2.4. Data Collection Methods

#### 2.4.1. Demographic Data and Academic Performance

Demographic data included the following: age, gender, marital status, specialty and academic year, smoking, consumption of caffeinated drinks, and confirmed physical or psychological illness. Participants were also asked to provide their GPA scores as a measure of academic performance both at end of the summer period and the academic term in which they were interviewed.

#### 2.4.2. Sleep Assessment

Participants were asked to complete four questionnaires including the Pittsburgh Sleep Quality Index (PSQI) for the assessment of sleep quality [27]. The PSQI measures seven components of sleep: 1. subjective sleep quality; 2. sleep latency (i.e., how long an individual takes to fall asleep); 3. sleep duration; 4. actual hours asleep; 5. sleep disturbances (i.e., having trouble sleeping); 6. use of sleeping medication, and 7. daytime dysfunction. Each sleep component was scored from 0 (no difficulty) to 3 (severe difficulty). The component scores were added together to provide an overall score (range: from 0 to 21). Higher scores indicate worse sleep quality. Scores greater than 5 indicate poor sleeping quality, as previously reported in the literature [27].

#### 2.4.3. Mental Health Assessments

Mental well-being was assessed with the following instruments: patient health questionnaire (PHQ)-9 for depression [28], general anxiety disorder (GAD)-7 for anxiety [29], Kessler Psychological Distress Scale (KPDS) for stress [30], Social Media Disorder Scale (SMD) for social media use [31].

#### 2.4.4. Working Memory Assessment

Working memory was assessed with CANTAB Cognitive Research Software Cambridge Cognition, n.d. All tests using CANTAB were standardized, computerized, and presented on a touch screen and have been widely used in numerous studies [32]. The spatial working memory (SWM) task was selected to assess working memory and required approximately 4 min to complete. Briefly, a number of boxes was displayed on the screen. Participants selected boxes and used their own strategy of elimination to find the yellow “token” in one of the boxes and to use the tokens to fill up an empty column on the right side of the screen. The test difficulty increased based on the number of boxes and could reach a maximum of 12 boxes. The token color and position inside the boxes changed from trial to trial to discourage the use of stereotyped search strategies. Working memory in the present study was measured by two fundamental factors: strategies and error measures. Strategies refer to the methods an individual can use to solve a CANTAB challenge, whereas error refers to the number of lost trials.

#### 2.4.5. Data Analysis

This analysis was conducted in two parts. First, the bivariate Pearson correlations represent the high-level associations between variables pertaining to sleep, working memory and mental well-being. These bivariate correlations do not control for one another, and may therefore be confounded by one another. This is why regression modeling was subsequently used to control for all measures. Multiple regression modeling investigated the association between GPA and the partial effects of all other variables (sleep, cworking memory, and mental well-being) while controlling for one another.

Scores were calculated for each of seven PSQI domains including sleep quality, latency, duration, efficiency, disturbances, the use of medications for sleep, and daytime dysfunction. Individual scores were added together to obtain a total PSQI score. Participants with scores of 5 or higher were considered poor sleepers [27]. In addition to sleep, we recorded SWM scores in terms of strategy scores (lower is better, for each trial containing 6, 6–8, or 6–12 tokens) and the total number of errors (for trials containing 4, 6, 8, 4–8, or 12 tokens).

For statistical modeling, each PSQI domain was coded as either adequate (0–1) or problematic (2–3), as were total scores (0–10 vs. 11–21). At the time of each visit, linear regression modeling was used to identify associations between GPA, sleep problems, and working memory, while controlling for anxiety, depression, and psychological distress. Level of education did not significantly contribute to the model and was not included as a covariate. We used multiple regression analysis to identify the partial associations between variables, in particular the potential overlap between measures (e.g., depression and anxiety). Multicollinearity among measures was tested by calculating the variance inflation factor (VIF) to ensure that all predictor variables could be modeled together and found that multicollinearity was negligible. We ensured a normal distribution of model residuals using QQ-plots. Statistical modeling was completed using R version 3.6.2.

## 3. Results

### 3.1. Participant Demographics and Sleep Characteristics

The sample included 83 participants (42.2% male) with a mean age of 21.82 years (SD = 1.59) at the time of the first visit. Sample demographics, sleep quality, and psychological scales are shown in Table 1. Two-tailed paired sample *t*-tests were used to compare these variables. Most participants in our study sample were medical students, n = 51 (61%). Global sleep scores were greater than 5 (poor sleepers) in 53 participants (64%) during the summer period, with the number increasing in the academic term to 64 participants (77%). There were significant differences between the two time points in overall PSQI scores, with worse scores at the academic term time point. These differences were also found in sleep duration, the need for medication, and daytime dysfunction. Significantly more psychological distress at the academic term time point was observed.

### 3.2. Working Memory Scores

Participant SWM scores are shown in Table 2. Two-tailed paired sample *t*-tests were used to compare SWM scores. Strategy scores for each type of trial were significantly lower in the academic term compared to the summer period. However, the total number of errors did not differ between these time points for any type of trial.

### 3.3. Mental Health Correlations with Sleep and Working Memory

We used Pearson correlation coefficients to assess which of the measures assessed showed significant associations before testing whether these correlations persist while controlling for other variables using linear regression. In the summer period, worse sleep quality overall was significantly correlated with worse symptoms of depression (r = 0.498), anxiety (r = 0.360), and distress (r = 0.433). These symptoms were specifically correlated with worse sleep quality, longer latency, more disturbances, and increased daytime dysfunction (Table 3). In addition, worse working memory strategy was correlated with increased sleep disturbances (6–12-token trials, r = 0.252), while more disordered social media use was significantly correlated with worse working memory strategy (6–12-token trials, r = 0.258) and more working memory errors (4–8-token trials, r = 0.249).

In the academic term, worse sleep overall was significantly correlated with increased symptoms of depression (r = 0.264), anxiety (r = 0.322), and distress (r = 0.400). These symptoms were all correlated with increases in sleep disturbances and worse daytime dysfunction. In addition, anxiety symptoms were correlated with longer sleep latency, and psychological distress was correlated with worse sleep quality. Worse SWM strategy was significantly correlated with having to take more medication for sleeping (6–12-token trials, r = −0.293), while more total SWM errors were significantly correlated with more problems with sleep duration (4–8 tokens, r = 0.246).

### 3.4. Association of Academic Performance with Sleep, Mental Well-Being, and Working Memory

**Figure 2:**
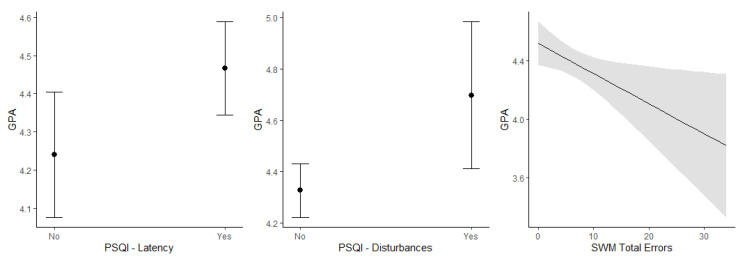
Significant associations between GPA and each component of PSQI (latency and disturbances) and working memory at academic term.

Linear regression analysis was used to assess the association between academic performance (measured as GPA) at end of the non-academic summer period and during the academic term, and partial effects of sleep quality as measured by PSQI (both the total and each of its components), distress, anxiety, and depression symptoms, as well as SWM strategy and total errors. The assessments were carried out once during the summer period and once during the academic term, and the findings are shown in Table 4. Briefly, GPA was not significantly associated with any sleep measures, psychological scales, or working memory in the summer period. In the academic term, higher GPA was significantly associated with longer sleep latency, more sleep disturbances, and fewer working memory errors (Figure 2). Moreover, in the academic term, a higher GPA was significantly correlated with better working memory strategy (6–12-token trials, r = −0.220) and fewer total errors (4–8-token trials, r = −0.247).

## 4. Discussion

The effect of sleep quality on academic performance has been previously investigated. Only a few studies, however, have examined the influence of mental well-being and working memory on this relationship, specifically, since working memory and mental health are well-known predictors of academic performance [33,34]. Therefore, our study aimed to expand the scope of previous investigations by using objective measures and a longitudinal design.

Our study revealed several interesting findings. First, we found that total sleep score, sleep duration, daytime dysfunction, and use of sleep medication worsened during the academic term compared to the non-academic summer period. Second, stress scores were significantly higher during the academic term, although other scores of mental health, such as depression and anxiety, numerically worsened as well. Third, despite worse sleep and mental health in the academic term, working memory scores improved in the academic term compared to the summer period, and working memory scores in the academic term were significantly correlated with a higher GPA. Moreover, longer sleep latency and increased sleep disturbances were also correlated with a high GPA in both the summer period and the academic term.

### 4.1. Sleep Habits among University Students

Most participants in our study were poor sleepers: 64% and 77% in the summer period and the academic term, respectively. This finding is consistent with recent reports of the increasing prevalence of poor sleep and a bid for better sleep hygiene practices among younger generations [35]. In addition, our findings show similar concordance with other national studies of sleep habits [24,25] along with international findings, including those in China [36] and Morocco [37]. Moreover, our study provides additional validation of previously reported results, as it assessed student sleep quality during two different time periods: the non-academic summer period and the academic term.

In the academic term, we found that students slept for a shorter duration, used more medication, and experienced more sleep disturbances and daytime dysfunction compared to that in the summer period. Sleep scores were better during non-academic term as students follow a more relaxed timeline, and although many of them may be still participating in community volunteering, research activities or elective rotations, students do not have set university exams or have to adhere to strict deadlines during the summer.

Such findings may be attributed to numerous stressors associated with academic life, such as the need to meet study deadlines, study intensively to achieve high grades, and keep up with social and family obligations [38]. Lifestyle behaviors affecting sleep patterns such as consumption of caffeinated or energy drinks [19] and excessive use of smartphones or social media platforms [23] may have contributed as well. Both behaviors (i.e., caffeine consumption and social media use) were also evident in our study population. These specific patterns in sleep disturbances may also signify increased anxiety, which has been previously described [37] among this age group, and may explain the increased use of sleep medication. Most importantly, this finding provides evidence that whichever lifestyle behaviors impair sleep hygiene, academic pressure further worsens such behaviors, which in turn negatively affect sleep quality.

### 4.2. Sleep Habits and Their Relationship to Academic Performance

The effect of sleep on the consolidation of memory is well known [37]. Previous work has demonstrated that a sleep-dependent neurophysiological process plays an essential role in the consolidation of various types of learning [39]. Similarly, disturbed sleep is associated with an overall decline in various aspects of cognition, which affects learning ability [40]. Previous studies have demonstrated a strong correlation between poor sleep quality and impaired academic performance [41,42,43]. Surprisingly, in our study, we found that students with a poorer sleep quality score (i.e., sleep latency and sleep disturbances) had a higher GPA during the academic term, whereas no relationship between sleep quality and GPA was observed in the summer period. The association between prolonged sleep latency and higher GPA could suggest that students were spending more time studying before falling asleep, which may explain the higher academic performance despite a pattern of deprived sleep. Similarly, a higher score for sleep disturbance associated with higher GPA could indicate that students with higher scores may worry more about their studies, hence their sleep is disturbed compared to low-achieving students. Interestingly, our study replicates the finding of another study conducted at the same university as the present study, which found that the prevalence of poor sleep quality among medical students was as high as 70.4%, especially among students with an excellent GPA [23]. A similar association between poor sleep and better academic performance was also documented among medical students in Saudi Arabia [24]. Furthermore, increasing evidence from the recent literature has suggested that good sleep and better academic performance do not always follow a linear pattern [44,45], although methodological differences in data collection and analysis may explain the discrepancy between conflicting results. It may be necessary to expand the simple model of a direct relationship between sleep quality and academic performance and explore other factors that potentially shape this relationship [26].

### 4.3. Working Memory, Academic Performance, and Sleep

Working memory, as justified in the introduction, is strongly linked to academic performance and equally vulnerable to sleep disruption. Furthermore, working memory is also a measure of executive function, not just memory, as it requires higher-order cognitive processing, e.g., retention and manipulation of information [12]. Spatial working memory specifically was previously investigated as more suspectable to sleep disruption then other forms of memory such as verbal working memory and declarative memory that are less affected by restricted sleep [46]. Working memory strategy scores were significantly lower (i.e., reflecting better working memory) during the academic term compared to the summer period. The improved cognitive functioning was evident despite worse sleep quality and mental health among our study sample in the academic term. Sleep is known to influence cognitive functions, including working memory [47], and previous work has found that sleep disturbances negatively impact working memory performance [47]. It should be noted, however, that the proposed impact of sleep quality on working memory is subject to individual differences, including age [48] and mood [49]. Working memory strategy scores improved from the summer period to the academic term, and the scores were correlated with GPA scores such that higher GPA was associated with better working memory performance, as reflected by a smaller number of errors and better strategy scores in the relevant task. Our findings of higher working memory scores, despite poor-quality sleep, may indicate that a student adopted a goal-oriented mindset during the academic term that focused on academic improvement and neglected well-being and physical needs, including sleep. This finding could be particularly evident in our sample, as the majority of the sample included students in highly competitive fields (i.e., medical students). Of particular interest is the fact that GPA did not reflect underlying global working memory (in the summer period) so much as the working memory state in a student can reach its maximum during the academic term.

### 4.4. Mental Health, Sleep, and Academic Performance

Younger generations in particular are increasingly exposed to stressors that have proven to negatively affect mental well-being [50]. This growing number of individuals suffering from the stress-induced effects of modern life may be the result of several factors, some of which include the increased use of social media [51] and peer relationships [52]. Increased stress among university students puts them at risk of developing mood disorders such as depression, which can not only negatively affect their capacity to learn, but also disrupt their sleep [35]. The student participants in the present study experienced mild anxiety, mild depression, and distress in both the non-academic summer period and the academic term. However, their distress scores significantly increased during the academic term. It has been previously reported that academic stressors predict depression, anxiety, and the number of sleep hours in students [53]. Hopelessness and anxiety have also been found to be mediators of academic stress and depressive symptoms [36]. Controlling these mental health mediators may help prevent academic-related stress and thereby improve wellbeing and sleep among university students [36].

In our study, we found poor sleep quality to be significantly correlated with poor mental health, and this correlation was evident in both the non-academic summer period and the academic term, specifically with regard to global sleep scores, but also sleep disturbances and daytime dysfunction. Previous work has demonstrated that sleep quality is correlated with mental health both locally and worldwide [25,54,55]. In our study, we also found that different sleep characteristics related to different mental health measures, thus potentially indicating different impact of sleep on mental health or vice versa but which cannot be inferred from an observational study like ours. Interestingly, it was previously reported that not only are sleep disorders prevalent among psychiatric disorders but also may by themselves increase the risk of such disorders developing later in life [56]. Taken together, these observations place great responsibility on academic institutions to raise awareness among university students on the importance of sleep hygiene, not only on physical but also mental health in the long term. Although we found that high GPA was correlated with poor sleep, and that poor sleep was further correlated with poor mental health, we did not find a direct association between psychological measures and GPA. These results do not accord with studies reporting that students with poor mental health have poor academic performance [57,58,59]. It is possible that high-achieving students, such as medical or health science students, may have traits that make them more resilient which may mask any underlying mental suffering. To confirm this supposition, e additional study with different populations would be required. In the same line of this finding, a study by Bemath et al. (2020) explored working memory in relation to psychological resilience among 38 healthy individuals. The study found that working memory indirectly fosters resilience-enabling behaviors [60]. Given that those students in our study with high GPA had a stronger working memory, it could be that such students are also capable of being more mentally resilient. That said, this is an important point to highlight, as such students are usually at high risk of developing burnout and possibly worse mental health outcomes, yet they may be overlooked because they are performing well academically [61].

### 4.5. Limitations

In the present study, we used a validated and self-reported measure to record sleep quality. This approach has limitations compared to more objective measures of sleep, such as polysomnography. Moreover, most of our participants were medical or health science students who may face higher levels of stress compared to other specialties and may be at higher risk of burnout in the future. This study only assessed spatial working memory. Other facets of cognition, including other forms of memory, may be either more or less associated with sleep, mental health, and academic performance. Finally, we focused on strategy scores as an outcome for spatial working memory performance with healthy volunters, and such a test is more likely to improve by practice effects when used repeatedly as in this study.

## 5. Conclusions

The findings from this study identify multiple factors that can potentially guide preventive and interventional programs to support the physical and mental health of university students. Despite the popular conception of the positive effect of sleep on academic performance, our results showed that high-achieving students were able to maintain a good GPA, which was correlated with better working memory, despite poor sleep quality. Academic institutions should direct resources to better educate, monitor, and support the students’ physical and mental health in the face of academic challenges, including those who are able to maintain good academic performance.

## Figures and Tables

**Table 1:** Participant demographics and characteristics at each time point. * signifies *p* < 0.05.

	Summer Period	Academic Term	Difference	Significance
Sample size	83	83		
Age in years—Mean (SD)	21.82 (1.59)	22.05 (1.37)		
Gender—Male—No. (%)	35 (42.2)	35 (42.2)		
Marital status—Single—No. (%)	81 (97.6)	80 (96.4)		
Smoker—Yes—No. (%)	10 (12.0)	12 (14.5)		
Caffeinated Drinks Yes—No. (%)	49 (92.5)	49 (96.1%)		
Confirmed illness—Yes—No. (%)	15 (18.1)	10 (12.0)		
Year number—Mean (SD)	4.36 (1.30)	4.43 (1.29)		
GPA—Mean (SD)	4.37 (0.53)	4.39 (0.46)	0.02	t (81) = −0.23, *p* = 0.816
PSQI—Total—Mean (SD)	6.99 (3.28)	7.98 (2.86)	0.99	t (82) = −2.64, *p* = 0.010 *
Quality score—Mean (SD)	1.25 (0.71)	1.34 (0.74)	0.08	t (82) = −0.93, *p* = 0.357
Latency score—Mean (SD)	1.58 (1.07)	1.58 (0.96)	0.00	t (82) = 0.00, *p* = 1.000
Duration score—Mean (SD)	1.00 (1.01)	1.51 (1.00)	0.51	t (82) = −3.93, *p* = 0.000 *
Efficiency score—Mean (SD)	0.74 (1.12)	0.57 (0.88)	−0.16	t (74) = 0.78, *p* = 0.439
Disturbances score—Mean (SD)	1.17 (0.50)	1.11 (0.47)	−0.06	t (74) = 1.15, *p* = 0.254
Medication score—Mean (SD)	0.27 (0.75)	0.47 (0.87)	0.20	t (82) = −2.06, *p* = 0.043 *
Dysfunction score—Mean (SD)	1.14 (0.91)	1.42 (0.89)	0.28	t (82) = −2.33, *p* = 0.023 *
Distress (K10)—Mean (SD)	22.27 (7.55)	25.63 (8.68)	3.36	t (82) = −4.01, *p* = 0.000 *
Anxiety (GAD-7)—Mean (SD)	6.94 (5.13)	7.86 (4.75)	0.92	t (82) = −1.63, *p* = 0.107
Depression (PHQ-9)—Mean (SD)	7.59 (6.23)	8.75 (5.77)	1.16	t (82) = −1.71, *p* = 0.091
Social Media (SMD)—Mean (SD)	2.60 (1.93)	2.24 (1.90)	−0.36	t (82) = 1.68, *p* = 0.097

**Table 2:** Participant spatial working memory scores at each time point. * signifies *p* < 0.05.

	Summer Period	Academic Term	Difference	Significance
**SWM Strategy**				
6-token trials	3.17 (0.96)	2.87 (1.16)	−0.3	t (82) = 2.35, *p* = 0.021 *
6–8-token trials	7.42 (2.18)	6.66 (2.72)	−0.76	t (82) = 3.01, *p* = 0.003 *
6–12-token trials	13.64 (3.75)	12.52 (4.58)	−1.12	t (82) = 2.83, *p* = 0.006 *
**SWM Total Errors**				
4-token trials	0.35 (0.76)	0.31 (0.85)	−0.04	t (82) = 0.40, *p* = 0.694
6-token trials	1.63 (2.64)	1.60 (2.88)	−0.02	t (82) = 0.07, *p* = 0.944
8-token trials	4.72 (5.68)	5.04 (5.93)	0.31	t (82) = −0.55, *p* = 0.582
4–8-token trials	6.70 (7.48)	6.95 (8.10)	0.25	t (82) = −0.36, *p* = 0.721
12-token trials	25.30 (15.03)	22.64 (14.84)	−2.66	t (82) = 1.25, *p* = 0.217

**Table 3:** Correlations between psychological scales, social media use, working memory, and PSQI components. Asterisks (*) indicate significance (*p* < 0.05).

Academic Term						
Measure	Distress	Anxiety	Depression	Distress	Anxiety	Depression
Total	r = 0.433 *	r = 0.360 *	r = 0.498 *	r = 0.400 *	r = 0.322 *	r = 0.264 *
Quality	r = 0.418 *	r = 0.221 *	r = 0.389 *	r = 0.253 *	r = 0.213	r = 0.184
Latency	r = 0.395 *	r = 0.235 *	r = 0.438 *	r = 0.143	r = 0.263 *	r = 0.136
Duration	r = −0.099	r = −0.063	r = −0.050	r = 0.151	r = −0.010	r = 0.056
Efficiency	r = −0.035	r = −0.031	r = 0.098	r = −0.057	r = −0.007	r = 0.006
Disturbances	r = 0.540 *	r = 0.608 *	r = 0.513 *	r = 0.385 *	r = 0.307 *	r = 0.370 *
Medication	r = 0.091	r = 0.178	r = 0.063	r = 0.144	r = 0.175	r = −0.012
Dysfunction	r = 0.630 *	r = 0.499 *	r = 0.644 *	r = 0.442 *	r = 0.241 *	r = 0.274 *
SWM Strategy	r = −0.126	r = −0.042	r = −0.034	r = −0.007	r = −0.123	r = 0.028
SWM Errors	r = 0.027	r = 0.01	r = 0.114	r = 0.148	r = −0.041	r = 0.098

**Table 4:** Association between sleep quality measures and GPA in the summer period and the academic term. Terms denoted with an asterisk (*) are significant (*p* < 0.05).

	Summer Period	Academic Term
PSQI—Total	F (1,62) = 0.13, *p* = 0.719	F (1,67) = 0.21, *p* = 0.646
Quality	F (1,62) = 0.42, *p* = 0.521	F (1,67) = 0.99, *p* = 0.323
Latency	F (1,62) = 2.81, *p* = 0.099	F (1,67) = 4.47, *p* = 0.038 *
Duration	F (1,62) = 3.98, *p* = 0.050	F (1,67) = 2.81, *p* = 0.098
Efficiency	F (1,62) = 1.60, *p* = 0.210	F (1,67) = 0.56, *p* = 0.456
Disturbances	F (1,62) = 0.51, *p* = 0.478	F (1,67) = 5.45, *p* = 0.023 *
Medication	F (1,62) = 0.41, *p* = 0.522	F (1,67) = 3.76, *p* = 0.057
Dysfunction	F (1,62) = 3.08, *p* = 0.084	F (1,67) = 0.04, *p* = 0.834
Distress (K10)	F (1,62) = 1.27, *p* = 0.263	F (1,67) = 0.54, *p* = 0.464
Anxiety (GAD-7)	F (1,62) = 0.28, *p* = 0.601	F (1,67) = 0.22, *p* = 0.642
Depression (PHQ-9)	F (1,62) = 1.03, *p* = 0.314	F (1,67) = 1.41, *p* = 0.239
SWM Strategy	F (1,62) = 1.11, *p* = 0.296	F (1,67) = 0.27, *p* = 0.602
SWM Errors	F (1,62) = 0.03, *p* = 0.868	F (1,67) = 5.50, *p* = 0.022 *

## Data Availability

Data available upon request due to ethical restrictions.
